# Perioperative and anesthesia-related cardiac arrests in geriatric patients: a systematic review using meta-regression analysis

**DOI:** 10.1038/s41598-017-02745-6

**Published:** 2017-06-01

**Authors:** Karen S. Braghiroli, José R. C. Braz, Bruna Rocha, Regina El Dib, José E. Corrente, Mariana G. Braz, Leandro G. Braz

**Affiliations:** 1 Department of Anesthesiology, Universidade Estadual Paulista (Unesp), Medical School, Botucatu, Brazil; 2 Department of Biostatistics, Universidade Estadual Paulista (Unesp), Institute of Biosciences, Botucatu, Brazil

## Abstract

The worldwide population is aging, and the number of surgeries performed in geriatric patients is increasing. This systematic review evaluated anesthetic procedures to assess global data on perioperative and anesthesia-related cardiac arrest (CA) rates in geriatric surgical patients. Available data on perioperative and anesthesia-related CA rates over time and by the country’s Human Development Index (HDI) were evaluated by meta-regression, and a pooled analysis of proportions was used to compare perioperative and anesthesia-related CA rates by HDI and time period. The meta-regression showed that perioperative CA rates did not change significantly over time or by HDI, whereas anesthesia-related CA rates decreased over time (P = 0.04) and in high-HDI (P = 0.015). Perioperative and anesthesia-related CA rates per 10,000 anesthetic procedures declined in high-HDI, from 38.6 before the 1990s to 7.7 from 1990–2017 (P < 0.001) and from 9.2 before the 1990s to 1.3 from 1990–2017 (P < 0.001), respectively. The perioperative CA rate from 1990–2017 was higher in low-HDI than in high-HDI countries (P < 0.001). Hence, a reduction in anesthesia-related CA rates over time was observed. Both perioperative and anesthesia-related CA rates only decreased with a high-HDI between time periods, and perioperative CA rates during 1990–2017 were 4-fold higher with low- compared to high-HDI in geriatric patients.

## Introduction

The number of elderly individuals is increasing more rapidly than that of other age groups^1^. By 2050, the number of people aged 60 years old and older is expected to be 2 billion, whereas it was 900 million in 2015^2^. Therefore, the number of geriatric patients who undergo anesthetic and surgical procedures will continue to increase. Additionally, the elderly experience a higher incidence of perioperative cardiac arrest (CA) than other adult age groups^3–10^.

Perioperative and anesthesia-related CA rates may be good indicators that can be used to analyze a country’s socioeconomic and health development over time, and it is advisable to explore the differences between hospital facilities for anesthetic and surgical procedures across different countries^11, 12^. A country’s developmental status is assigned by the Human Development Index (HDI)^13^, a national development status index based on enrollment in higher education, literacy, per-capita income, and life expectancy. As previously described^14, 15^, high- and low-income countries are defined as those having an HDI ≥ 0.8 and <0.8, respectively. A previous meta-analysis including patients in all age groups revealed a decreased in the risk of both perioperative and anesthesia-related mortality rates over the last five decades, especially in high-HDI countries^14^. A recent systematic review involving patients in all age groups employed meta-analysis to show that there was a reduction in perioperative and anesthesia-related CA rates in high-income countries and an increase in perioperative CA rates, but with no change in the anesthesia-related CA procedures, in low-income countries when comparing pre-1990s with 1990–2014^15^. In this aforementioned study, anesthesia-related and perioperative CA rates were found to be reduced with increasing HDI status but not with time by meta-regression analysis.

Although there is a consensus that anesthesia and surgery are now safer than they were in the past and that more geriatric patients are undergoing these procedures^16^, no systematic review using meta-regression and regarding worldwide rates of perioperative and anesthesia-related CA events in geriatric patients is available in the literature. We hypothesized that perioperative and anesthesia-related CA rates have decreased over time in high- and low-HDI countries by performing a weighted meta-regression and weighted CA rates for geriatric patients published in cohort and cross-sectional studies.

The current study aimed to perform the first meta-regression analysis of the worldwide data on perioperative and anesthesia-related CA rates in geriatric patients over time and according to national HDI values. Additionally, we examined pooled weighted CA rates of perioperative and anesthesia-related CA events in low- and high-HDI countries during the pre-1990s and the 1990–2017 period.

## Results

### Selection of studies

**Figure 1:**
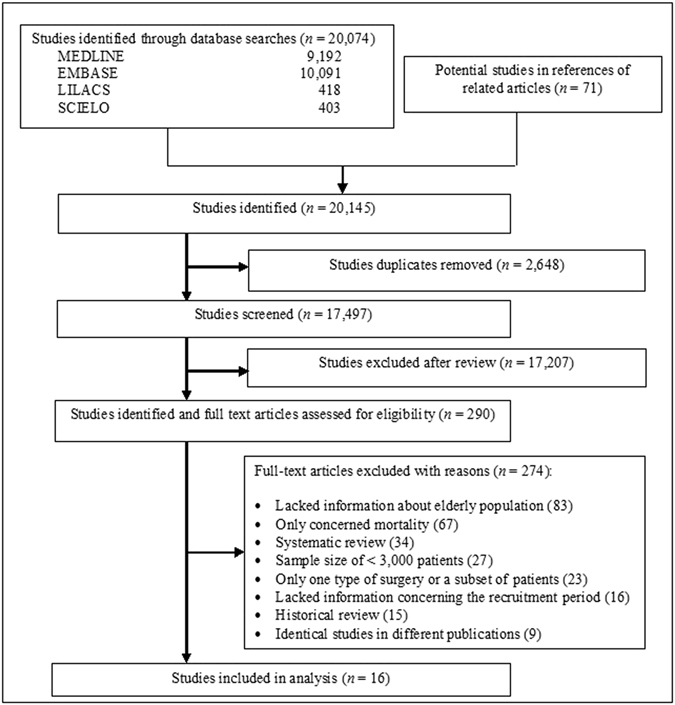
Flowchart of the process used to identify studies for inclusion.

Our literature search yielded 20,074 citations in addition to 71 potential studies that were identified in the references of related articles. We excluded 2,648 duplicate studies, and after reviewing the titles and abstracts, we retrieved 290 potentially relevant full-text articles for more detailed evaluation. Of these articles, 16 studies met the inclusion criteria (Fig. 1).

### Study characteristics

Sixteen studies from nine countries fulfilled the inclusion criteria. These studies involved 1,758,153 anesthetic procedures performed during surgery in geriatric patients. The characteristics and designs of these studies are listed in Table 1.

**Table 1:** Characteristics of included studies with references.

Investigator and year of publication	Data source and study period	Median year	HDI average	Primary outcome	Weight %	Cardiac arrest	Patients	Excluded	Age group included
Ahmed *et al*.^39^ 2008	Single University Hospital Audit – Pakistan 1992–2006	1999	0.527	Cardiac arrest in OR and PACU	1: 20.3	1: 14	32,742	Cardiac surgery	≥60 years
Aubas *et al*.^3^ 1991	Single University Hospital Chart review – France 1983-1987	1985	0.974	Cardiac arrest in OR and PACU	2: 21.3	2: 8	8,432	—	≥75 years
Biboulet *et al*.^4^ 2001	Single University Hospital Database – France 1989–1995	1992	0.960	Cardiac arrest within 12 hours	2: 7.5	2: 4	7,544	ASA V patients	≥75 years
Braz *et al*.^40^ 1999	Tertiary University Hospital Database – Brazil 1988–1996	1992	0.759	Cardiac arrest in OR and PACU	1: 19.7	1: 35	6,982	—	≥65 years
Braz *et al*.^7^ 2006	Tertiary University Hospital Database – Brazil 1996–2005	2001	0.767	Cardiac arrest in OR and PACU	1: 19.7, 2: 27	1: 48, 2: 4	6,796	—	≥65 years
Dam & Vimtrup^31^ 1967	Single Hospital Database – Denmark 1955–1965	1960	0.971	Cardiac arrest in OR	1: 25.0	1: 14	12,737	—	≥70 years
Deiner *et al*.^10^ 2014	Multicentric - University and Community Hospitals Database – USA 2010–2013	2012	0.908	Cardiac arrest within 48 hours	1: 25.6	1: 557	972,505	—	≥65 years
Fiscella *et al*.^41^ 1991	Single Private Hospital Prospective survey – Argentina 1980–1990	1985	0.953	Cardiac arrest within 24 hours	1: 24.5	1: 15	5,473	—	> 60 years
Goswami *et al*.^8^ 2012	Multicentric - 304 Hospitals Prospective survey – USA 2005–2007	2006	0.953	Cardiac arrest in OR	1: 24.0	1: 130	80,834	Cardiac surgery Trauma cases	≥70 years
Kawashima *et al*.^6^ 2002	Multicentric - 467 Hospitals Questionnaire – Japan 1999	1999	0.928	Cardiac arrest within 7 days	1: 25.1, 2: 45.2	1: 224, 2: 21	208,568	—	> 65 years
Kubota *et al*.^42^ 1994	Tertiary University Hospital Database – Japan 1962–1992	1977	0.989	Cardiac arrest in OR	1: 25.1	1: 1	15,351	Cardiac surgery, Organ transplantation	≥65 years
Morita *et al*.^43^ 2002	Multicentric - 536 Hospitals Questionnaire – Japan 2000	2000	0.933	Cardiac arrest within 7 days	1: 25.2, 2: 47.3	1: 282, 2: 22	272,734	—	≥65 years
*Nunes *et al*.^16^ 2014	Tertiary University Hospital Database - Brazi1 1996–2010	2003	0.716	Cardiac arrest in OR and PACU	1: 20.2, 2: 73.0	1: 100, 2: 6	18,367	—	≥60 years
Olsson & Hallén^5^ 1988	Single Hospital Database – Sweden 1976–1984	1980	0.987	Cardiac arrest in OR	2: 40.8	2: 43	60,563	—	> 60 years
Otteni *et al*.^44^ 1986	Multicentic - 460 Hospitals Prospective survey – France 1978–1982	1980	0.974	Cardiac arrest within 24 hours	1: 25.3, 2: 37.8	1: 252, 2: 50	39,620	—	≥60 years
*Tamdee *et al*.^17^ 2009	Single University Hospital Database – Thailand 2003–2007	2005	0.780	Cardiac arrest within 24 hours	1: 19.9	1: 36	8,905	Cardiac surgery	≥65 years

*Abbreviations*. HDI: Human Development Index, the score ranges from 0 to 1, which represents the lowest and highest levels of development, respectively; OR: operating room; PACU: postanesthesia care unit; Weight (%): study weight contribution to the pooled analysis of the proportion effect size; 1: perioperative cardiac arrest; 2: anesthesia-related cardiac arrest; *Studies that included only geriatric patients.

### Meta-regression analysis

**Figure 2:**
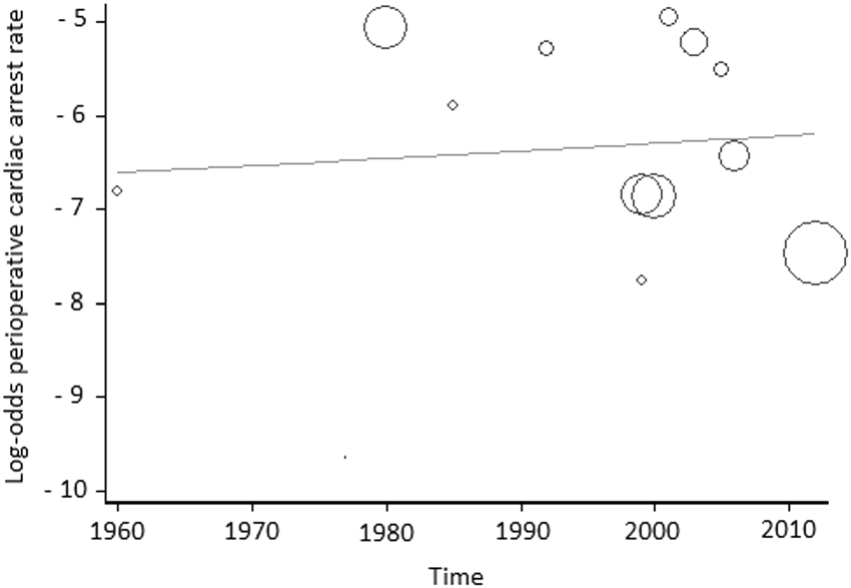
Meta-regression of perioperative cardiac arrest rates according to time. Each circle represents a study and indicates its weight in the analysis. The correlation between perioperative cardiac arrests and time was not significant (slope: 0.0079; 95% CI: −0.0483 to 0.0642; P = 0.76).

**Figure 3:**
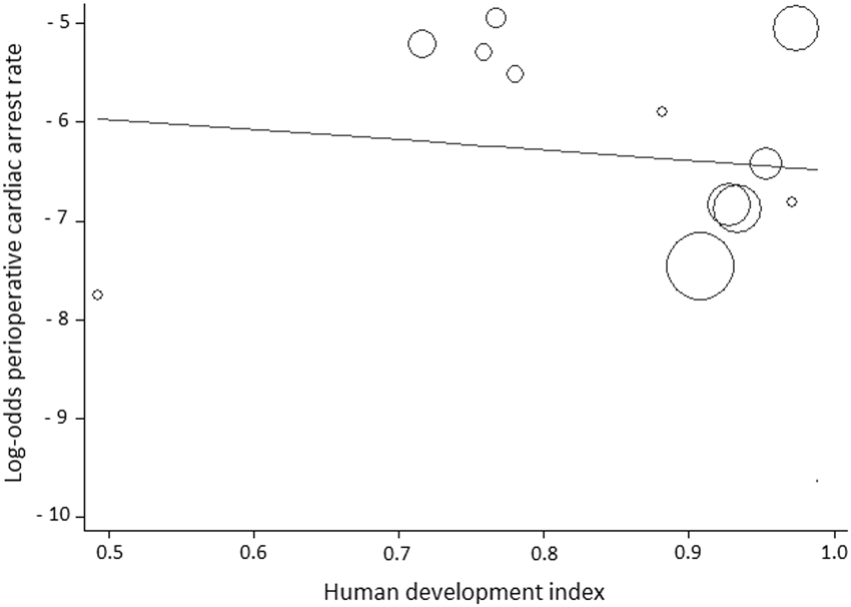
Meta-regression analysis of perioperative cardiac arrest rates according to Human Development Index (HDI) status. Each circle represents a study and indicates its weight in the analysis. The correlation between perioperative cardiac arrests and HDI was not significant (slope: −1.0389; 95% CI: −6.7380 to 4.6601; P = 0.69).

**Figure 4:**
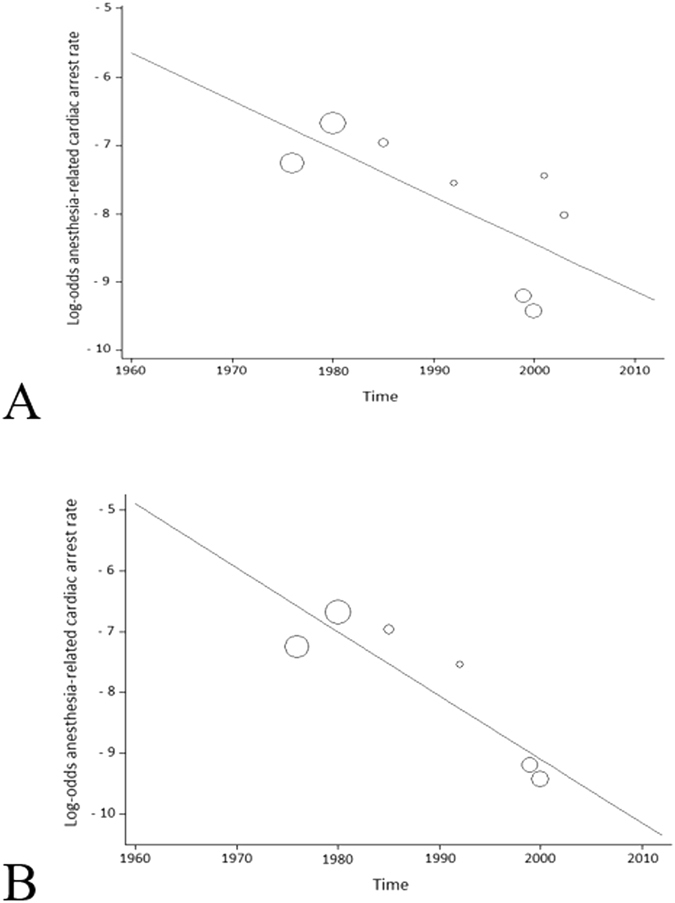
Meta-regression of anesthesia-related cardiac arrest rates according to time. Each circle represents a study and indicates its weight in the analysis. (**A**) The correlation between anesthesia-related cardiac arrests and time was significant considering both low- and high-HDI countries (slope: −0.0699; 95% CI: −0.1394 to −0.0003; P = 0.04). (**B**) The correlation between anesthesia-related cardiac arrests and time was significant in only high-HDI countries (slope: −0.1049; 95% CI: −0.1762 to −0.0336; P = 0.015).

**Figure 5:**
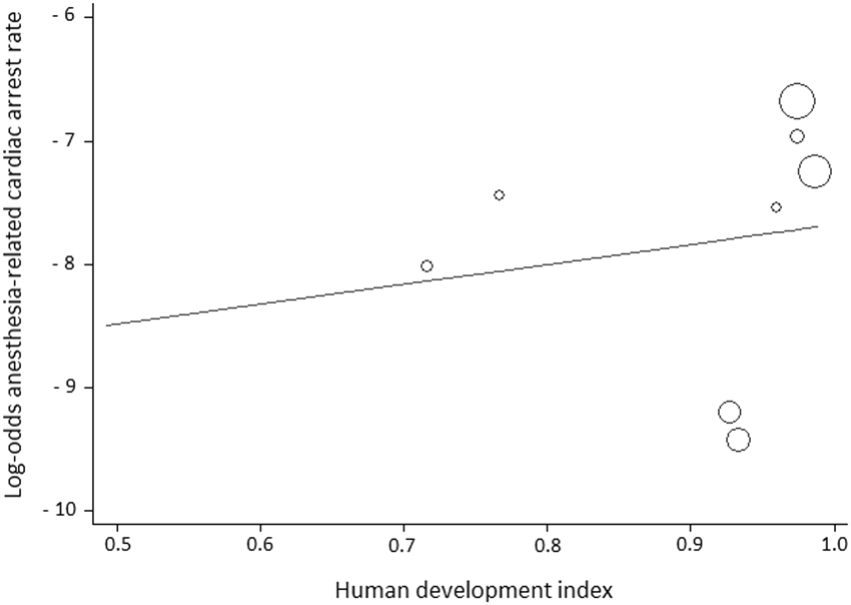
Meta-regression analysis of anesthesia-related cardiac arrest rates according to Human Development Index (HDI) status. Each circle represents a study and indicates its weight in the analysis. The correlation between anesthesia-related cardiac arrests and HDI was not significant (slope: 1.6188; 95% CI: −8.6021 to 11.8399; P = 0.71).

Of the evaluated studies (*n* = 16), 13 were related to perioperative CA (Figs 2 and 3), and eight were related to anesthesia-related CA (Figs 4 and 5).

#### Time (study year)

The data from all of countries assessed in the weighted meta-regression showed no statistically significant relationship between perioperative CA and time (slope: 0.0079; 95% CI: −0.0483 to 0.0642; P = 0.76; Fig. 2). However, there was a significant decrease in the anesthesia-related CA rate over time, regardless of HDI status (slope: −0.0699; 95% CI: −0.1394 to −0.0003; P = 0.04; Fig. 4A). There was also a significant decrease in this rate over time in high-HDI countries (slope: −0.1049; 95% CI: −0.1762 to −0.0336; P* = *0.015; Fig. 4B). Correlation between anesthesia-related CA rates and time was not calculated for low-HDI countries because these rates were described in only two articles, which made the meta-regression analysis impossible.

#### HDI status

The weighted meta-regression analysis revealed no relation between the perioperative CA rate and HDI status (slope: −1.0389; 95% CI: −6.7380 to 4.6601; P = 0.69; Fig. 3). Similarly, there was no significant correlation between the anesthesia-related CA rate and a country’s HDI (slope: 1.6188; 95% CI: −8.6021 to 11.8399; P = 0.71; Fig. 5).

### Pooled weighted analysis of CA rate

When we compared studies to determine whether a country’s HDI status had an effect on CA events during the two evaluated time periods (pre-1990s *versus* 1990–2017), we found that in high-HDI countries, perioperative CA rates decreased from 38.6 (95% CI: 37.8–39.6) per 10,000 procedures before the 1990s to 7.7 (95% CI: 7.6–7.8) per 10,000 procedures in 1990–2017 (P < 0.001); anesthesia-related CA rates decreased from 9.2 (95% CI: 8.9–9.5) per 10,000 procedures before the 1990s to 1.3 (95% CI: 0.2–6.5) per 10,000 procedures in the 1990–2017 (P < 0.001; Table 2). None of the studies evaluated perioperative or anesthesia-related CA rates in low-income countries before the 1990s, making sub-analysis of data for these periods impossible.

**Table 2:** Perioperative and anesthesia-related cardiac arrests by time period and by human development index (HDI) status.

	Studies	*I* ^2^	Events	Patients	Pooled weighted event rate per 10,000 anesthetic procedures (95% CI)	P Value for Subgroup		
						High- *vs* low-HDI	High-HDI per time period*	Low-HDI per time period*
**Perioperative cardiac arrest**								
Pre-1990s	4		282	73,181		NA		
High-HDI	4	98.9	282	73,181	38.6 (37.8–39.6)		<0.001	
Low-HDI	NR	NR	NR	NR	NR			NA
1990–2017	9		1,426	1,608,433		<0.001		
High-HDI	4	98.0	1,193	1,534,641	7.7 (7.6–7.8)			
Low-HDI	5	98.0	233	73,792	31.3 (30.4–32.2)			
**Anesthesia-related cardiac arrest**								
Pre-1990s	3		101	108,615		NA		
High-HDI	3	73.9	101	108,615	9.2 (8.9–9.5)		<0.001	
Low-HDI	NR	NR	NR	NR	NR			NA
1990–2017	5		57	514,009		0.57		
High-HDI	3	71.8	47	488,846	1.3 (0.2–6.5)			
Low-HDI	2	NA	10	25,163	4.6 (0.1–264.9)			

*Abbreviations*. *I* ^2^: heterogeneity among studies; CI: confidence interval; NR: not reported; NA: not available; *vs*: *versus*; *Pre-1990s *versus* 1990–2017.

In the period from 1990 to 2017, perioperative CA rates were 4-fold lower in high-HDI countries [7.7 per 10,000 procedures (95% CI: 7.6–7.8)] than in low-HDI countries [31.3 per 10,000 procedures (95% CI: 30.4–32.2; P < 0.001; Table 2)], though there were no significant differences between low- [4.6 per 10,000 procedures (95% CI: 0.1–264.9)] and high-HDI countries [1.3 per 10,000 procedures (95% CI: 0.2–6.5; P = 0.57; Table 2)] concerning anesthesia-related CA rates.

For all event rates, the proportion of heterogeneity (*I* ^*2*^) varied from 71.8% to 98.9% (Table 2).

## Discussion

In this systematic review of surgeries in geriatric patients, we used meta-regression analysis to show that perioperative CA rates did not change over time or with regard to HDI status; in contrast, anesthesia-related CA rates did significantly decrease over time but not according to HDI status. In addition, by using the pooled weighted CA rate analyses, we found that both perioperative and anesthesia-related CA rates were lower in high-HDI countries in the later time period. However, it was not possible to analyze changes in these event rates in the low-HDI countries because there was a lack of published studies covering the period before the 1990s. During the 1990–2017 period, perioperative CA rates were 4-fold lower in high-HDI than in low-HDI countries.

Based on these findings, the following question arose: Why did perioperative CA rates not decline over time or by HDI status in geriatric patients? A previous meta-regression analysis of data obtained from a systematic review of anesthesia-related and perioperative mortalities in high- and low-income countries showed that the baseline American Society of Anesthesiologists (ASA) physical status of patients has increased significantly in recent decades^14^. In the same study, meta-regression by ASA status was performed to evaluate CA events and deaths, and a significantly increasing relationship was found between a higher ASA status at baseline and CA events or deaths^14^. Additionally, studies evaluating low- and high-HDI countries have demonstrated that geriatric patients who present a poorer ASA physical status (III–V) are more likely than ASA I–II patients to suffer perioperative CA or death^16–19^.

Elderly individuals are more predisposed than young people to cardiovascular disease. For example, a ruptured aneurysm or complications associated with cardiac surgery or intraoperative myocardial infarction are important causes of intraoperative CA and death in geriatric patients^16^. Therefore, the baseline risk for and complexity of managing geriatric patients undergoing surgery are both increased. Geriatric patients, many of whom are frail, can experience considerable comorbidities and cognitive impairment and have reduced physical activity, all of which are associated with perioperative complications^20^. These factors are thought to contribute to the significant increasing correlation between perioperative CA rates and time. However, we found no significant change in this relationship, possibly indicating that increased perioperative safety measures are implemented with regard to geriatric patients.

The significant reduction we observed in anesthesia-related CA rates over time may be associated with considerable improvements in anesthesia safety since the early 1990s, especially in high-HDI countries. We can also attribute this finding to several safety improvements, such as advancement in drugs, training programs, guidelines and checklists, investments in monitoring techniques, especially the capnograph and pulse oximeter, and systematic approaches to reduce errors^21–23^. According to Eichhorn, these initiatives, which are aimed at achieving “safety monitoring”, have resulted in a continuous increase in intraoperative vigilance and improved patient safety during anesthesia^24^. In our study, the relationship between anesthesia-related CA rates and time could not be evaluated in low-income countries because only two relevant articles were identified. This made performing meta-regression analysis using these data impossible.

The results of our pooled weighted CA rate analyses, which were divided into HDI status and time period, demonstrated the existence of a considerable gap between the healthcare systems of low- and high-income countries. For example, in high-HDI countries, there were significant 5- and 7-fold reductions in perioperative and anesthesia-related CA rates, respectively, between the two time periods. Additionally, our study showed that the perioperative CA rate was 4-fold lower in high-HDI than in low-HDI countries from 1990 to 2017. Although the anesthesia-related CA rate was also 3.5-fold higher in low-HDI countries than in high-HDI countries during this period, this difference was not significant. Regardless, this result may have been influenced by the relatively small number of both studies and patients that were identified for low-HDI countries.

Several studies have highlighted the major role that pre-anesthetic management of a patient’s condition plays in minimizing complications and adverse outcomes^18, 25–28^. One study performed in a low-income country showed that several geriatric patients have poor health when undergoing surgery^16^. These findings demonstrate that there is a need for improving the quality and quantity of resources that can be used as well as access to healthcare, both of which are inadequate, in developing countries. Additionally, it is necessary to adopt perioperative medical practices that have demonstrable effectiveness, to organize multidisciplinary discussions of adverse effects, to implement evidence-based safety protocols, to provide better methods for selecting patients for surgery, and to initiate advances in techniques, protocols, and pre- and postoperative critical care to improve perioperative patient care^29, 30^.

There are some data limitations in the current study. One of the main limitations is that several papers were excluded from the review due to the lack of information about both the numbers of geriatric patients and the patients who developed CA in this specific age group. Additionally, many of the included papers presented different age group subdivisions, including both adult and geriatric patients who developed CA (50–69^8^; 15–69^31^; 55–74 years^3, 4^); this only enabled the inclusion and analysis of patients ≥70 or ≥75 years, respectively, in the aforementioned studies. There were also differences across the included studies regarding surgical populations (e.g., excluded ASA V patients), time frames (e.g., intraoperative, 24 hours or 48 hours postoperatively, or seven postoperative days), and procedures (e.g., exclusion of organ transplantation, trauma, or cardiac surgery). Accordingly, these differences contributed to the high heterogeneity observed in our study. A random-effects model was applied to minimize this heterogeneity when assessing trends between the two time periods and the two HDI status categories. In addition to including ≥3,000 geriatric patients, we also calculated the weighted rate of CA events across all studies to minimize possible bias. The last limitation is that there were no pre-1990s studies performed in low-income countries that either fulfilled the inclusion criteria or were not published in an indexed journal, and the absence of such studies may have contributed to publication bias.

We evaluated a public health problem that is associated with the geriatric population. This age group experiences a considerable number of diseases and functional dependencies that contribute to higher health system costs and a substantial impact on economic, social, and family dynamics^32^. It is therefore necessary to expand and intensify our research into aging and evidence-based medicine to provide better geriatric care in high-HDI and, in particular, low-HDI countries^33^. Indeed, there is a large disparity between the healthcare systems of these countries, and our study showed that improvements have been made in geriatric patient safety care during anesthetic procedures in high- but not in low-income countries. As explained by Koga *et al*., future efforts and collaborations should involve improvements in perioperative safety to reduce the gap between high- and low-HDI countries in their respective health care systems^15^. Further reviews of perioperative and especially anesthesia-related CA must be periodically performed to obtain continued worldwide CA rates in geriatric patients in low- and high-HDI countries. We also showed that there was a decrease in anesthesia-related CA rates over time while there was an increase in the size of the geriatric population and the associated number of comorbidities.

In conclusion, our meta-regression analysis shows that perioperative CA rates did not change over time or based on a country’s HDI; conversely, anesthesia-related CA rates decreased over time but not according to HDI. By using pooled weighted CA rate analyses, a notable reduction in perioperative and anesthesia-related CA rates between the two time periods was observed in high-HDI countries. In addition, perioperative CA rates were 4-fold lower in high- than in low-income countries during the 1990–2017 period.

## Methods

This manuscript was prepared in accordance with Meta-analyses Of Observational Studies in Epidemiology statements (MOOSE)^34^. According to the local institutional review board (IRB), there was no need for Ethical approval due to the type of study.

### Search Strategy and Inclusion and Exclusion Criteria

The following databases were searched until April 2^nd^, 2017: EMBASE, MEDLINE, LILACS, and SCIELO. Additionally, we manually reviewed all potential studies and included relevant articles. No restrictions were applied for year of publication or language.

The literature search was performed using MeSH terms and text words including synonyms about perioperative and/or anesthesia-related fatal and non-fatal CA rates in geriatric patients. To achieve higher sensitivity and to identify relevant articles, the search strategy was adjusted for each database (see Supplementary S1.1 and S1.2).

The following inclusion criteria were applied for the studies: (i) included patients aged 60 years and older and reported perioperative and/or anesthesia-related CA rates; (ii) involved all age groups, including patients aged 60 years old and older; (iii) a cohort or cross-sectional study; (iv) specified perioperative and/or anesthesia-related CA rates that occurred seven days after the surgery; and (v) provided enough information to perform the analysis.

The exclusion criteria were as follows: (i) reported only one surgical procedure (e.g., cardiac surgery) or a specific anesthetic technique (e.g., regional anesthesia) or patient subtype (e.g., patients with ASA physical status I and II only); (ii) did not specify the time period; or (iii) evaluated fewer than 3,000 geriatric patients. At least 3,000 patients in each study were included to allow us to estimate the incidence of rare adverse events (≤1 per 1,000 anesthetics)^35^.

### Data Extraction and Outcome Definitions

Two investigators (K.S.B and L.G.B.) identified studies, and consensus was reached in all cases.

The primary outcome was perioperative CA (an event resulting from any cause; e.g., patient disease/condition, surgery, or anesthesia). The secondary outcome was anesthesia-related CA (either partly or completely attributable to anesthesia) by the investigators of the included studies. Because there is no consensus regarding the definition of the postoperative period (in the worldwide literature, the definitions of postoperative period include the first 24 hours, 48 hours, and even 1 month after surgery), the period of perioperative CA was defined in this review as the date of surgery until the seventh postoperative day.

High- and low-income countries were defined as having an HDI ≥ 0.8 and <0.8, respectively. A country’s HDI status can change over time, and several studies have reported data covering a time period of many years; thus, HDI was defined as the mean of HDI values between the first and last year in which the patients were recruited for each study. When HDI was not available for the specific time period covered by the study, we used the HDI for the closest available date.

Due to the fact that many safety-improvement measures emerged in the early 1990s in high-HDI countries and later in some low-HDI countries and based on two systematic reviews of perioperative CA^14, 15^, the time frame evaluated in the studies included in this analysis was split into two periods (pre-1990s *versus* 1990–2017). These measures included improvements in the care of patients, which involved equipment sterilization, new anesthesia medications and workstations with ventilators, anesthesia-practice based on safety protocols, and an increase in the number of adult intensive care beds^14, 15, 21–23^.

### Statistical Analysis

Our meta-regression analysis used a fixed-effect model with restricted estimated maximum likelihood (REML) and an observed log-odds ratio to predict whether CA rates in geriatric patients statistically changed over time or according to HDI (time and HDI were evaluated as continuous variables). Stata-13 (StataCorp LP, College Station, TX) was used to perform the meta-regression.

Additionally, a random-effects model was applied to calculate weighted event rates across all of the included studies with a pooled analysis (StatsDirect Ltd, Altrincham, Cheshire, UK)^36^. The times and HDI values were divided (i.e., pre-1990s *versus* 1990–2017 and low-income *versus* high-income, respectively) to evaluate perioperative and anesthesia-related CA rates. The event rate was defined as the number of CA events per 10,000 anesthetic procedures, and the data are reported as 95% confidence intervals (CIs). Proportion tests were performed using SAS for Windows^®^, v.9.4 (SAS Institute, Cary, NC). Chi-square tests were used to compare differences in the proportions of events according to time period and HDI status. Because the data were recorded as time intervals (e.g., from 06/06/1989 to 06/06/1993), the median year of the study’s patient recruitment interval (i.e., median: 1991)^15^ was considered. A P value < 0.05 was considered significant.

An alternative approach that is employed to quantify the effect of heterogeneity is the *I* ^2^ statistic (StatsDirect Ltd, Altrincham, Cheshire, UK)^37, 38^; values higher than 50% suggest heterogeneity among the studies.

## Electronic supplementary material

Supplementary Information
