# Supplementary material for: Perioperative and anesthesia-related cardiac arrests in geriatric patients: a systematic review using meta-regression analysis
Source: Sci Rep. 2017 Jun 1;7:2622. doi: 10.1038/s41598-017-02745-6 (PMC5453984; doi:10.1038/s41598-017-02745-6)
Supplement: Supplementary file 1 — Supplementary Information [file 41598_2017_2745_MOESM1_ESM.pdf]

Perioperative and anesthesia-related cardiac arrests in geriatric patients: a systematic review using meta-regression analysis

Karen S. Braghiroli[1], José R. C. Braz[1], Bruna Rocha[1], Regina El Dib[1], José E. Corrente[2], Mariana G. Braz[1] & Leandro G. Braz[1]

Supplementary Information

S1.1 Web Search Strategy for MEDLINE and EMBASE (up to April 2nd, 2017)
(Anaesthesia-related mortality OR Anesthesia-related mortality OR Anaesthesia-related cardiac arrest OR Anesthesia-related cardiac arrest OR ((cardiac arrest OR heart arrest OR Asystole OR Asystoles OR Cardiopulmonary arrest) **AND** (anaesthesia OR anesthesia))) **AND** (cross-sectional OR cross-sectional study OR cross-sectional studies OR crossectional study OR crossectional studies OR cross sectional study OR cross sectional studies OR prospective OR retrospective OR prospective design OR retrospective design OR prospective study OR prospective studies OR retrospective study OR retrospective studies OR case series OR review)

S1.2 Web Search Strategy for SCIELO AND LILACS (up to April 2nd, 2017)
(anesthesia OR anaesthesia) **AND** (cardiac arrest OR mortality OR death)
